# Spreading Topsoil Encourages Ecological Restoration on Embankments: Soil Fertility, Microbial Activity and Vegetation Cover

**DOI:** 10.1371/journal.pone.0101413

**Published:** 2014-07-01

**Authors:** Desirée Rivera, Violeta Mejías, Berta M. Jáuregui, Marga Costa-Tenorio, Ana Isabel López-Archilla, Begoña Peco

**Affiliations:** 1 Departamento de Ecología, Universidad Autónoma de Madrid, Madrid, Spain; 2 Dirección Técnica, OHL Construcción, Madrid, Spain; 3 Departamento de Biología Vegetal I, Universidad Complutense, Madrid, Spain; NERC Centre for Ecology & Hydrology, United Kingdom

## Abstract

The construction of linear transport infrastructure has severe effects on ecosystem functions and properties, and the restoration of the associated roadslopes contributes to reduce its impact. This restoration is usually approached from the perspective of plant cover regeneration, ignoring plant-soil interactions and the consequences for plant growth. The addition of a 30 cm layer of topsoil is a common practice in roadslope restoration projects to increase vegetation recovery. However topsoil is a scarce resource. This study assesses the effects of topsoil spreading and its depth (10 to 30 cm) on two surrogates of microbial activity (β-glucosidase and phosphatase enzymes activity and soil respiration), and on plant cover, plant species richness and floristic composition of embankment vegetation. The study also evaluates the differences in selected physic-chemical properties related to soil fertility between topsoil and the original embankment substrate. Topsoil was found to have higher values of organic matter (11%), nitrogen (44%), assimilable phosphorous (50%) and silt content (54%) than the original embankment substrate. The topsoil spreading treatment increased microbial activity, and its application increased β-glucosidase activity (45%), phosphatase activity (57%) and soil respiration (60%)**.** Depth seemed to affect soil respiration, β-glucosidase and phosphatase activity. Topsoil application also enhanced the species richness of restored embankments in relation to controls. Nevertheless, the depth of the spread topsoil did not significantly affect the resulting plant cover, species richness or floristic composition, suggesting that both depths could have similar effects on short-term recovery of the vegetation cover. A significant implication of these results is that it permits the application of thinner topsoil layers, with major savings in this scarce resource during the subsequent slope restoration work, but the quality of topsoil relative to the original substrate should be previously assessed on a site by site basis.

## Introduction

The construction of road, railway and other transport-related civil engineering infrastructure has greatly increased in recent decades. It has severe impacts on ecosystems, including habitat fragmentation, soil compaction, heavy erosion and sediment transport [1]–[3]. Topographic changes such as the creation of totally new artificial slopes are also associated with this engineering infrastructure. The angle, compaction and erosion potential of the new slopes hinder the rapid establishment of vegetation, which makes restoration enhancement necessary [4], especially in arid and semiarid conditions and in soils with low organic matter content [5], [6]. Road slope colonization by plants is constrained by several factors such as seed dispersal capacity, slope type (roadcuts and embankments), aspect and soil physic-chemical properties [7], [8]. Techniques such as hydroseeding, topsoil spreading, geotextile installation and planting [9]–[12] are used to encourage plant cover on roadslopes. Soil properties and vegetation cover can be improved by spreading topsoil [13] due to its content in seeds, plant propagules, nutrients and microorganisms which restore soil functionality [9], [14]. The top 30 cm of soil is removed routinely at the start of road construction works. This substrate is stockpiled for the restoration project, and at the end of the works, a 30 cm layer of stockpiled topsoil is spread over the embankments. Few studies have analysed the effects of this restoration technique on soil microbial activity [15] and other soil fertility-related properties [13], despite the fact that topsoil is a scarce resource at many linear infrastructure construction sites, and issues such as the optimum topsoil depth to be spread during roadslope restoration have yet to be resolved.

Plant-soil interactions have a major influence on the structure and function of terrestrial ecosystems, increasing or decreasing the speed of plant succession and/or its trajectories [16]–[18]. To date, restoration management has mainly focused on manipulating succession in plant communities [18], although ecosystem restoration enhancement also requires consideration of the metabolic capacity of the soil (henceforth, soil functionality).

Several studies of primary succession in soils affected by heavy stress [18], [19] have shown the importance of microorganisms in the restoration of soils and plant communities. Soil microbes are largely responsible for the cycling of elements within the soil [20] and are important drivers of plant diversity and productivity in terrestrial systems [21]. In disturbed areas, vegetation establishment depends on the effectiveness of the microorganisms to increase the availability of nutrients in the soil through the decomposition of organic matter, and also their ability to establish symbiotic relationships [22], [23].

Soil functionality can be addressed through surrogate variables such as enzyme activity, related to the nutrient cycle, soil respiration and other physic-chemical properties related to soil fertility [24], [25]. The most widely tested soil enzymes are those involved in the degradation of cellulose, the major components of plant litter, such as β-glucosidase [26], [27], which makes it a good indicator of organic matter [28] and thus of the C, N, P and S available to plants [29]. β-glucosidase is also an effective predictor of organic C accumulation in soil in Mediterranean environments [30]. Extracellular phosphatases are also of interest due to their role in mineralizing P from nucleic acids, phospholipids and other ester phosphates [31]. Moreover, soil enzyme activity, particularly that of β-glucosidase and phosphatase, is a good indicator of the state of terrestrial ecosystems due to their sensitivity to changes in soil management [27], [32]. On the other hand, soil respiration is the sum of root respiration and the decomposition of soil organic matter, plant litter, and root exudates by soil microorganisms. Soil surface CO_2_ flux is an indirect measurement of the soil respiration rate, under the assumptions that the CO_2_ flux is at a steady state and that all CO_2_ flux is a result of respiration. This parameter provides a good measurement of the ecosystem's overall metabolism and indirectly, of microbial activity as well [33], [34]. Additional soil properties such as pH, organic matter, N-P-K content and texture are also widely used parameters of soil fertility [35].

This study assesses the effects of topsoil spreading and depth on soil microbial activity, measured as enzymatic activity and soil respiration, plant cover, species diversity and species composition on a railway embankment in Central Spain. It also evaluates the difference between topsoil and the original embankment substrate in selected physic-chemical properties related to soil fertility.

We tested the following hypotheses: i) topsoil has higher fertility than the original embankment substrate, ii) topsoil spreading enhances enzymatic activity and soil respiration in disturbed soils, iii) added topsoil changes plant cover, species diversity and floristic composition on the embankments, and iv) the effects of topsoil spreading on microbial activity and vegetation can be modified by changing the depth of the topsoil added in restoration projects.

## Material and Methods

### Study area

The study area was a railway line under construction in the vicinity of Dehesa de Mari Martin, Navalcarnero, west of Madrid, Spain (40° 18 'N, 3° 58' W), located on tertiary sands resulting from the erosion of the granitic Sierra de Guadarrama Mountains. The area has a slightly undulating relief, with a 20 year old *Pinus pinaster* plantation as the predominant vegetation.

The local semiarid Mediterranean climate has cold winters, hot summers and summer drought. Average temperatures in the coldest and warmest months are 9 and 20°C respectively, with 425 mm average annual rainfall.

### Experimental design

A recently built north-facing railway embankment with a 14° slope was selected for the experiment. A randomized 5 block design with three treatments was applied to 15 adjacent plots measuring 6×9 m. The treatments were: control (no added topsoil), T10 (addition of a 10 cm deep topsoil layer) and T30 (addition of a 30 cm deep topsoil layer).

Treatment T30 reflected current slope restoration practices, while T10 was selected for three reasons: a) to reduce erosion on the slopes, b) to reduce seed loss which might be occurring in the bank due to burial, given that few seeds can germinate and/or reach the surface from depths lower than 5 cm [36], and c) to optimize the topsoil, a scarce resource in great demand for linear infrastructure construction.

The topsoil used for the experiment was collected from the uppermost 30 cm of soil in the first week of December 2009 in a nearby pine forest through which the railway line will pass. Topsoil collection occurred after the summer drought and before significant autumn rains, and after the removal of woody vegetation (shrubs and trees). The topsoil was stockpiled for two days and spread on the experimental plots immediately afterwards.

### Topsoil and soil fertility

To characterize the physic-chemical soil properties of the original embankment substrate and the stockpiled topsoil, 1 kg soil samples were randomly collected from the embankment (n = 15) and topsoil stockpiles (n = 15) prior to spreading, using eight 4 cm diameter by 10 cm deep soil cores for each composed sample. Samples were dried and sieved through a 2 mm pore diameter mesh, and analysed for their content in organic matter, total nitrogen, assimilable phosphorus and potassium, percentages of sand, silt and clay, and pH. The organic matter (OM) was analysed following the protocol described by Walkley and Black [37]. Total nitrogen was measured with the Kjeldahl method, and assimilable P and K were extracted with acetate-EDTA as per Läkanen and Erviö [38] and Cottenie et al. [39], respectively. The percentages of soil fractions (sand, silt and clay) were measured by the hydrometer method as per Day [40]. Finally, a 1:2.5 soil/water solution was used for the soil pH analysis.

### Enzymatic activity

Six months after the experiment started (June 2010), at the time of expected maximum enzymatic activity due to the temperature and soil moisture conditions, soil samples were collected from the experimental plots (control, T10 and T30) with two replicate of soil cores (4 cm diameter by 10 cm depth), in three slope positions: the upper, middle and lower parts of each plot. We used 10 cm deep soil cores to study soil enzymatic activity, as other authors have done previously in semiarid Mediterranean grasslands [25], [41], where annual vegetation roots are usually restricted to the first 10 cm below the soil surface. The collected material was air dried, sifted through a 2 mm pore diameter sieve and then analysed for phosphatase and β-glucosidase enzyme activity.

Phosphatase acid (EC 3.1.3.2) was determined colorimetrically as the amount of p-nitrophenol released per 0.5 g of soil after being incubated for 1 hour at 37°C with a solution of p-nitrophenyl phosphate substrate in a MUB pH 6.5 buffer [42]. The same method described for the phosphatase was used to determine the β-glucosidase (EC 3.2.1.21), with a p-nitrophenyl β-D-glucopyranoside substrate in a THAM pH 6 buffer [43], [44].

For both enzymes, the spectrophotometric absorbance was measured in a Varian Cary 1C UV Spectrophotometer at 400 nm. The amount of p-nitrophenol produced in each sample (enzyme activity) was defined using calibration curves obtained from the patterns at different concentrations of p-nitrophenol, for phosphatase and β-glucosidase.

### Soil respiration

Soil basal respiration was determined as the rate of CO_2_ production, measured with an infrared gas analyser coupled to a flow chamber (IRGA, EGM-4 Environmental Gas Monitor for CO_2_). The chamber was placed in three slope positions (upper, middle and lower) in each of the experimental plots, delimited by partially buried PVC pipes. Random measurements of soil respiration were taken in each plot once every month between from July 2010 and June 2011, between 10:00 and 16:00 h to avoid bias due to daily variations in temperature or soil moisture. Aboveground vegetation in the delimited areas was removed at least one week prior to the in situ measurements.

### Plant cover, species diversity and composition of roadslope vegetation

The cover of each plant species was determined visually in spring 2010 and 2011, the optimum period for studying Mediterranean herbaceous communities. This involved setting up six 0.5 m quadrats in each plot (control, T10 and T30) in two slope positions, three quadrats set 1 m away from the edge of the upper plot and the other three 1 m from the edge of the lower plot. The total percentage cover of herbaceous plants was noted and two indicators of species diversity (species richness (S) and equitability (E = H'/ln S), [45]) were calculated.

### Data analysis

The differences in the physic-chemical soil properties of topsoil vs. original embankment substrate was analysed with ANOVAs. β-glucosidase and phosphatase enzyme activity were analysed using a univariate general linear model (GLM). We produced a model for each parameter in which net enzyme activity (µmol of p-nitrophenol g^−1^·h^−1^) was included as the dependent variable, with the treatment (control, T10 and T30) and slope position (upper, middle, lower) and their interaction as fixed factors. The differences between factor levels were analysed with post-hoc Tukey tests. Mean soil respiration data (CO_2_ production rate in g CO_2_·m^−2^·h^−1^) per plot obtained during the twelve sample months was analysed with a repeated measures General Linear Model. The CO_2_ production rate was the within-subject factor. Levels were months (12 measures) and the treatment (control, T10 and T30) and slope position (upper, middle, lower) were the between-subjects factors. Finally, total plant cover (square root transformed), species richness (log transformed) and equitability, were analysed by means of a repeated measures ANOVA, with the year as the within-subjects variable, and treatment (control, T10 and T30) and slope position (upper, lower) as the between-subjects factors. Where their interaction proved significant, a year by year ANOVA was performed and differences between factor levels were analysed by a Tukey test. For all statistical analysis we used SPSS 15.0 software (SPSS Inc., Chicago, IL, USA).

The response of floristic composition to the treatments was tested by Redundancy Analysis (RDA) using the CANOCO 4.5 program [46] and CANODRAW for Windows 4.12 [47] for the graphics. As environmental variables we used treatment, slope position and year. Forward automatic selection with Monte Carlo test (499 permutations) was used to find the environmental factors that best explained species cover values. We evaluate the final model with a Monte Carlo permutation test. The percentage cover values for species were log-transformed.

To compare the effects of treatments on vegetation over time, the Principal Response Curves method (PRC) was employed, using CANOCO (versión 4.5; [48]). Previously an RDA was carried out, introducing treatment interactions with years as environmental variables (excluding the interaction of period with the control which served as a reference series) and the year as covariable (in application terms). The statistical significance of this ordination was tested with a Monte Carlo permutations test (499 permutations). The significance of a second axis, when the first proved significant, was analysed with another RDA introducing the sample coordinates in the first axis as a covariable, in addition to the year [49].

### Ethics Statement

Prior to the field studies, we obtained all the permits for the works. The Site Manager of Obrascón Huarte Lain S.A. and Madrid Regional Government were informed, since experimental plots were located on an embankment under construction. No protected species were sampled during the study.

## Results

### Topsoil and soil fertility

Most of the physic-chemical measured soil properties showed significant differences between topsoil and original embankment substrate (Table 1). Topsoil had significantly higher soil organic matter, total nitrogen content, assimilable P and lower soil pH than the original embankment substrate. Non-significant differences were observed for assimilable K. The percentage of silt was also higher in topsoil than in the original substrate, but there were no significant differences for percentage of clay or sand (Table 1).

**Table 1:** Physic-chemical parameters (means ± SE) of original embankment substrate and topsoil used for the experiment.

**Parameters**	**Original substrate**	**Topsoil**	***F***	***P***
Available water (g/kg)	58.0±6.2^b^	41.4±6.8^a^	16.36	0.04
OM (g/kg)	1.2±0.8^a^	10.4±1.8^b^	107.47	<0.001
P (mg/100 g)	0.2±0.1^a^	0.4±0.1^b^	7.36	0.03
N (g/kg)	0.2±0.0^a^	0.4±0.1^b^	29.92	0.001
K (mg/100 g)	6.2±0.6	5.7±0.6	1.84	0.21
pH	6.4±0.1^b^	5.8±0.0^a^	152.81	<0.001
Clay (g/kg)	169.3±13.0	105.4±2.7	5.21	0.06
Sand (g/kg)	722.3±22.6	694.9±73.5	0.63	0.45
Silt (g/kg)	108.4±12.6^a^	199.7±12.9^b^	127.19	<0.001

OM: Organic matter; P: phosphorus content; K: potassium content; N: nitrogen content. Different letters (a, b) indicate significant differences (*P*>0.05) between treatments.

### Spread topsoil and microbial activity

**Figure 1:**
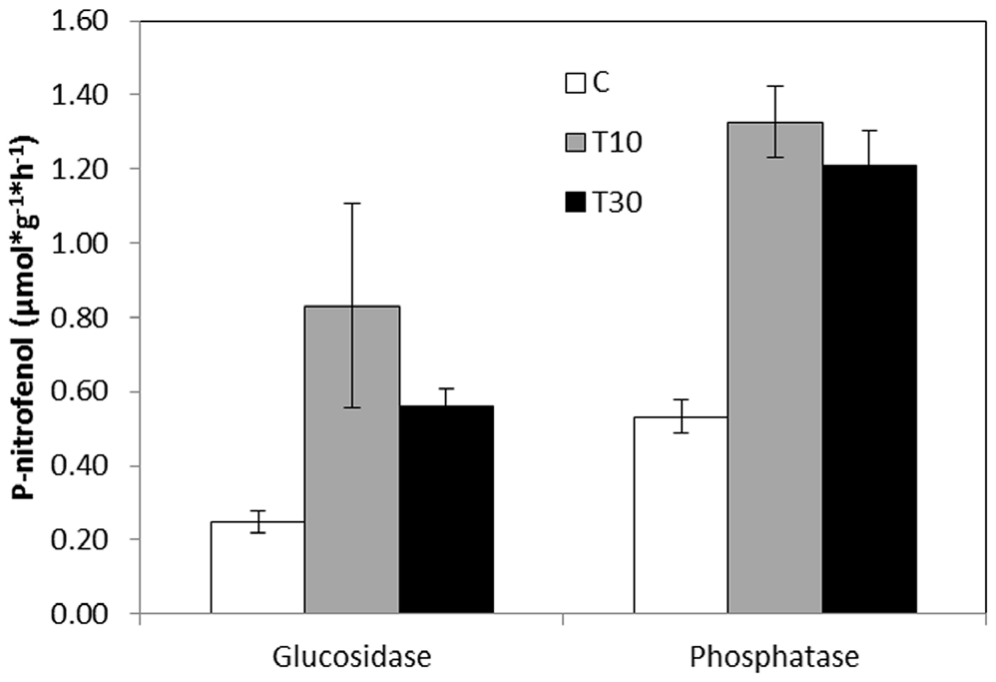
Enzymatic activity with topsoil. β-Glucosidase and Phosphatase activity in soil with added topsoil. C: control; T10: 10 cm deep layer of spread topsoil and T30: 30 cm deep layer of spread topsoil. Bars show standard error.

Both of the tested enzymes, β-glucosidase and phosphatase, showed higher activity in the plots with spread topsoil than in the control plots (F = 17.91, *P*<0.001 for β-glucosidase and F = 35.73, *P*<0.001 for phosphatase) (Fig. 1.). Nevertheless, there were no significant differences in enzyme activity between topsoil depths (10 and 30 cm) (Post-hoc Tukey test, *P* = 0.69 for β-glucosidase and *P* = 0.79 for phosphatase). Differences in enzymatic activity according to soil location on the slopes (upper, middle and lower) were not significant for either β-glucosidase (F = 2.36, *P* = 0.084) or phosphatase (F = 1.60, *P* = 0.214).

**Figure 2:**
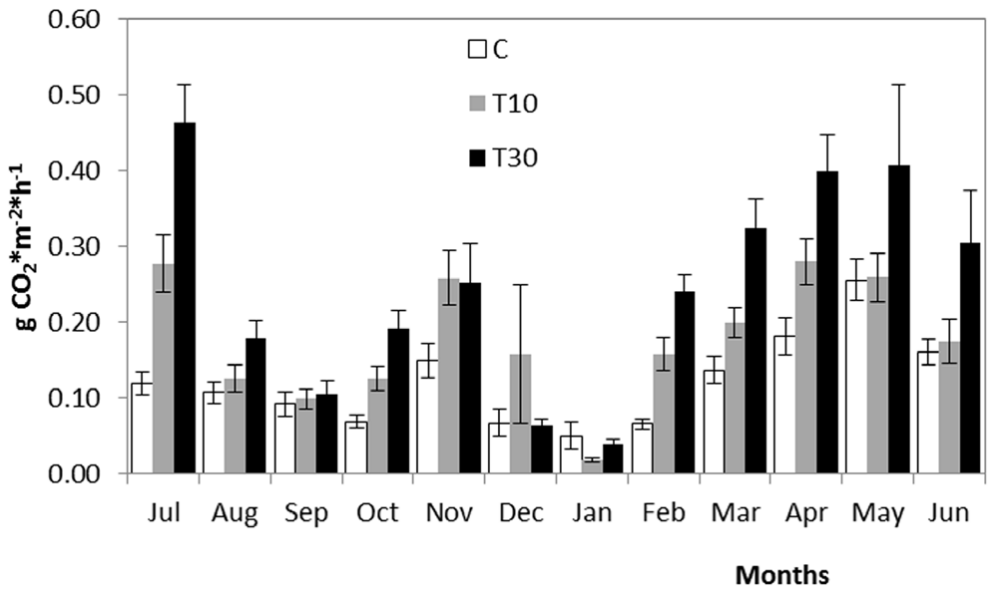
Annual CO_2_ production rate. Monthly mean and standard errors of CO_2_ production rate (g CO_2_·m^−2^·h^−1^) per treatment for the period July 2010-June 2011. C: control; T10: 10 cm deep layer of spread topsoil and T30: 30 cm deep layer of spread topsoil.

Soil respiration varied through the year (F = 98.47; *P*<0.001). The lowest values of soil respiration were recorded in the driest or coldest months (summer and winter), while the highest values corresponded to the period with most moisture and mildest temperatures (Fig. 2). The repeated measures analysis showed a significant effect of the treatment (F = 29.28; *P*<0.001), with a generally higher CO_2_ production in plots with the largest volume of fertile topsoil, T30 (0.23±0.022 g CO_2_·m^−2^·h^−1^), than in the control (0.12±0.011 g·CO_2_·m^−2^·h^−1^) and T10 treatments (0.17±0.017 g·CO_2_·m^−2^·h^−1^). In this case, plot position on the slope showed a significant effect on soil respiration (F = 4.26; *P*<0.05), with higher soil respiration in the lower position (0.19±0.018 g CO_2_·m^−2^·h^−1^) than in the upper or middle plots (0.15±0.014 and 0.18±0.018 g CO_2_·m^−2^·h^−1^, respectively). The interaction (treatment x position) was not significant (F = 6.35; *P*>0.05).

### Topsoil spreading and total plant cover, species richness and equitability

Total plant cover was higher in the control than in the topsoil plots during the first year. Such cover increased in all plots during the second year until it was equal in all treatments. The ‘period x treatment' interaction proved significant (F = 9.36; *P*<0.001; Table 2) since the increase of herbaceous cover over time was greater in the topsoil plots than in the controls. The depth of spread did not affect total plant cover in either study year (Table 2).

**Table 2:** Mean values (means ± SE) for total plant cover (%), species richness and equitability in spring 2010 and spring 2011 for each treatment.

Period	Treatment	Plant cover	Richness	Equitability
2010	Control	40.8±3.5^ b^	7.2±1	0.77±0.04^a^
	T10	21.7±2.0^a^	6.9±0.8	0.84±0.05^ab^
	T30	17.3±2.1^a^	6.2±1.3	0.85±0.09^b^
2011	Control	62.0±3.3	14.6±1.63^a^	0.83±0.03
	T10	52.2±3.8	17.8±1.95^b^	0.81±0.03
	T30	61.7±3.2	19.5±1.89^b^	0.82±0.03

a, b indicate significant differences (*P* < 0.05) between treatments within a year

T10  =  topsoil applied to 10 cm depth, T30  =  topsoil applied to 30 cm depth.

In total, 121 species were identified over the two years. Of these, 79 species were found in the control plots and 94 in the topsoil plots (Table S1). *Avena barbata, Capsella bursa-pastoris, Carlina corymbosa, Diplotaxis erucoides, Plantago lagopus, Psilurus incurvus, Trifolium glomeratum* and *Trifolium scabrum* occurred exclusively on the control plots. Although species richness was similar under all treatments during the first year, it was higher in the topsoil plots than in the controls in the second year (period x treatment interaction F = 10.46; *P*<0.001; Table 2). The depth of the spread topsoil did not affect species richness in either study year (Table 2).

Equitability was greater in the topsoil plots than in the controls during the first year (Table 2), but these differences disappeared during the second year (period x treatment interaction F = 3.6; *P* = 0.03). The depth of spread did not affect species equitability in either study year (Table 2).

Slope position was not significant in any of these models (F = 0.18; *P* = 0.67, for plant cover; F = 0.02; *P* = 0.90, for species richness and F = 0.13; *P* = 0.72, for species equitability).

### Topsoil spreading and floristic composition

**Figure 3:**
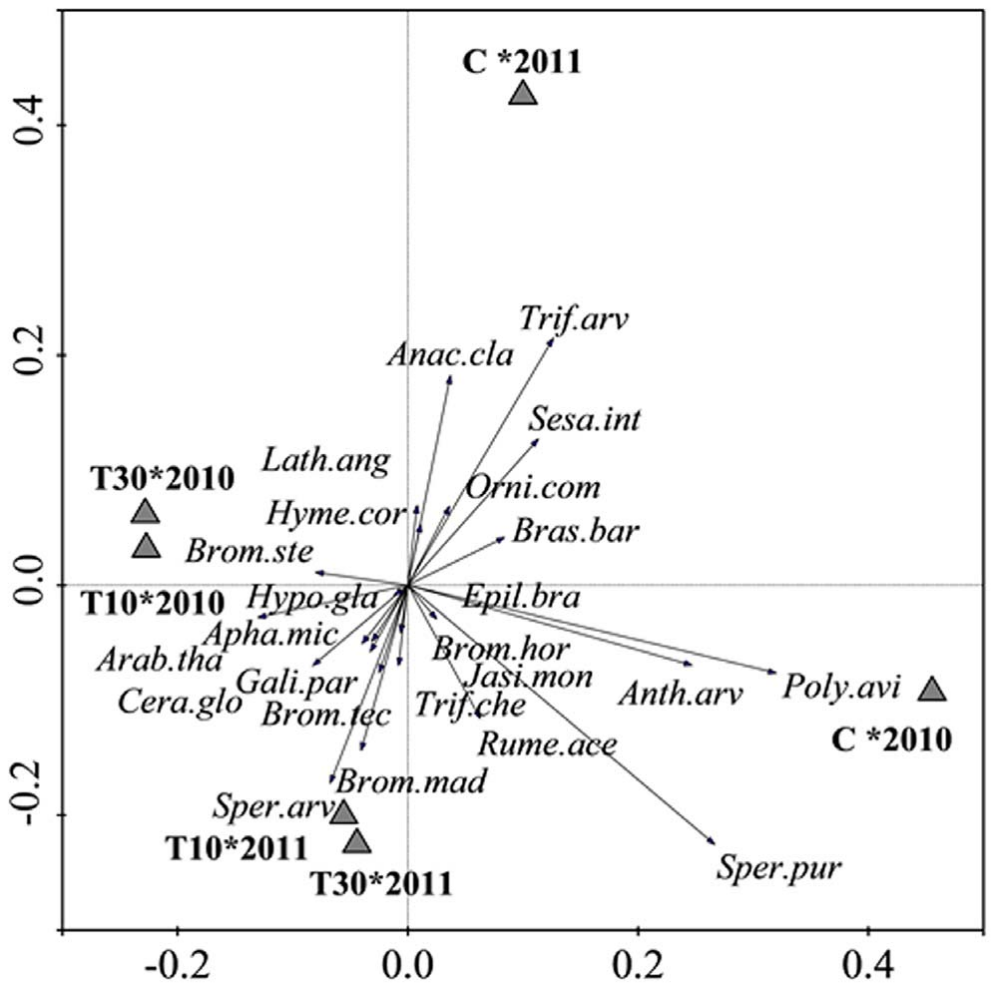
Floristic composition by year and treatment. RDA ordination diagram, first and second axes for species against treatments in each year (2010 and 2011). C, Control; T10, 10 cm topsoil depth; and T30, 30 cm topsoil depth. **Ana.cla.,** *Anacyclus clavatus*; **Anth.arv.,** *Anthemis arvensis*; **Apha.mic.,** *Aphanes microcarpa*; **Arab.tha.,** *Arabidopsis thaliana*; **Bras.bar.,** *Brassica barrelieri*; **Brom.hor.,** *Bromus hordeaceus*; **Brom.mad.,** *Bromus madritensis*; **Brom.ste.,** *Bromus sterilis*; **Brom.tec.,** *Bromus tectorum*; **Cera.glo.,** *Cerastium glomeratum*; **Epil.bra.,** *Epilobium brachycarpum*; **Gali.par.,** *Galium parisiense*; **Hyme.cor.,** *Hymenocarpus cornicina*; **Hypo.gla.,** *Hypochaeris glabra*; **Jasi.mon.,** *Jasione montana*; **Lath.ang.**, *Lathyrus angulatus*; **Orni.com.,** *Ornithopus compressus*; **Poly.avi,** *Polygonum aviculare*; **Rume.ace.,** *Rumex acetosella*; **Sesa.int.,** *Sesamoides interrupta*; **Sper.arv.,** *Spergula arvensis*; **Sper.pur.,** *Spergularia purpurea*; **Trif.arv.,** *Trifolium arvense*; and **Trif.che.,** *Trifolium cherleri*.

**Figure 4:**
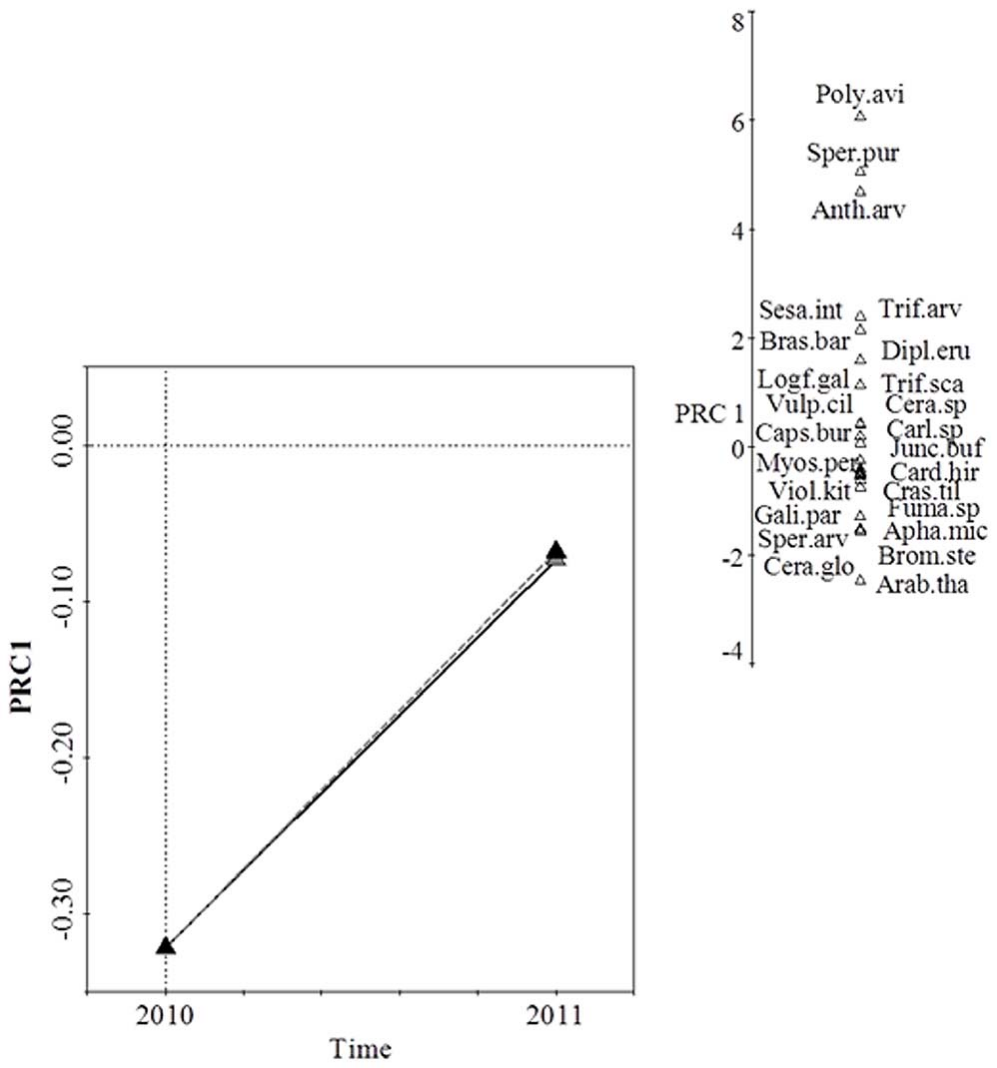
Species composition response to the depth of the spread topsoil. Diagram of the first (a) and second (b) PRC axes. The first two PRC axes proved significant (*F* = 13.09; *P* = 0.002 for axis 1 and *F* = 12.95; *P* = 0.002 for axis 2). The dotted line indicates the 10 cm topsoil treatment and the solid line the 30 cm treatment. In the ordination axis of the PRC diagram, the first component of the variation explained by the differences in treatment over time is represented, with time on the x axis. The weighting of each species on the principal axes, shown on the right of the PRC diagram, represents the affinity of each species to the treatments. Only species showing adjustments greater than 1 are included. **Anth.arv.,** *Anthemis arvensis*; **Apha.mic.,** *Aphanes microcarpa*; **Arab.tha.,** *Arabidopsis thaliana*; **Bras.bar.,** *Brassica barrelieri*; **Brom.ste.,** *Bromus sterilis*; **Caps.bur.,** *Capsella bursa-pastoris*; **Card.hir.,** *Cardamine hirsuta*; **Carl.sp.,** *Carlina sp*.; **Cera.sp.,** *Cerastium sp*.; **Cera.glo.,** *Cerastium glomeratum*; **Cras.til.,** *Crassula tillaea*; **Dipl.eru.,** *Diplotaxis erucoides* **Fuma.sp.,** *Fumaria* sp.; **Gali.par.,** *Galium parisiense*; **Junc.buf.,** *Juncus bufonius*; **Logf.gal.,** *Logfia gallica*; **Myos.per.,** *Myosotis personii*; **Poly.avi,** *Polygonum aviculare*; **Sesa.int.,** *Sesamoides interrupta*; **Sper.arv.,** *Spergula arvensis*; **Sper.pur.,** *Spergularia purpurea*; **Trif.arv.,** *Trifolium arvense*; **Trif.sca.,** *Trifolium scabrum*; **Viol.kit.,** *Viola kitaibeliana*; and **Vulp.cil**., *Vulpia ciliata*.

In the analyses of floristic composition, the environmental variables year and treatment were significant (F = 51.05; *P* = 0.002 and F = 12.82; *P* = 0.02, respectively) and were included in the model in the automatic selection procedure, while slope position was not significant (F = 1.37; *P* = 0.09) and was removed from the model. The first two RDA axes accounted for 83% of the species cover variation (Fig. 3), with 67% of the variation explained by the first axis. Floristic composition segregated during the first year (spring 2010) between the topsoil plots (T10–2010 and T30–2010) and the controls (C–2010). Three species, *Spergularia purpurea, Polygonum aviculare* and *Anthemis arvensis*, showed the highest cover values in the control plots during the first year, whereas those with highest cover values on the topsoil plots were *Bromus sterilis* and *Arabidopsis thaliana*. *Spergula arvensis* and *Bromus madritensis* had the highest cover values on the T10 and T30 plots in 2011, whereas *Trifolium arvense* and *Anacyclus clavatus* then provided the greatest cover on the control plots. The floristic composition on the T10 and T30 plots differed more from the control plots in the first year than in the second (Fig. 4). *Polygonum aviculare, Spergularia purpurea* and *Anthemis arvensis* showed the highest weightings on the first axis, in accordance with the RDA results (Fig. 3).

Species composition was not affected by the depth of the spread topsoil (Fig. 4). The ordination diagram of the second PRC axis did not provide any additional information to that of the first axis.

## Discussion

### Topsoil and soil fertility

According to our results, we can assume that the addition of topsoil to the original substrate in embankments will increase soil fertility and that this improvement will depend on the depth of the topsoil added to the original substrate. Organic matter content, total nitrogen and assimilable phosphorus were all higher in topsoil in comparison with the original embankment substrate. Organic matter was tenfold higher in the topsoil than in the original substrate, while N and P showed an increase of 50% (Table 1). This improvement was obviously linked to the quality of the spread topsoil, the depth from which it had been collected, the material used to build the embankments and the local climate. In more xeric conditions, Tormo et al. [13], for example, found more total N and assimilable P in plots with spread topsoil than without it, but found no differences in the organic matter content, which was very low in both cases (less than 1.5%). Similarly, the relatively low pH found in the analysis of the topsoil in the present experiment could be related to the origin of the material used in the experiment, a *Pinus pinaster* plantation, with many acidic compounds in the needles [50] which tend to acidify the soil.

### Topsoil spreading and microbial activity

The results of this study are, to our knowledge, the first empirical evidence that topsoil spread on new slopes shaped during the construction of linear transport infrastructure improves microbial activity and soil functioning, measured through surrogate parameters, such as the activity of enzymes involved in vital nutrient cycles and soil respiration.

Plots where topsoil was spread showed more β-glucosidase and phosphatase activity than in the control plots, with an increase of 45% and 57% respectively, which supports our initial hypothesis. The differences found between the controls and the plots with topsoil were probably due to the higher organic matter content in the latter (Table 1). Several authors have found a positive relationship between the activity of these enzymes with respect to soil organic C content [32], [51], [52]. Inorganic nutrients required for plant and microorganism growth need to be released from the organic matter. When soil microorganisms detect a nutrient shortage, they start up their internal machinery and produce exo-enzymes, which release nutrients in an inorganic form from the dead organic matter. The addition of topsoil increases microbial activity and ultimately benefits plant growth, especially if it is rich in organic matter. However, the absolute values of the activity of these enzymes were lower than those found by other authors in similar climates [18], [53]. This may be linked to the quality of the organic matter in the experimental topsoil, which was taken from a pine plantation and therefore may have contained a high proportion of extremely recalcitrant compounds [54], possibly limiting the microbial activity.

The observed decrease in soil pH after topsoil addition (Table 1) does not seem to have a negative effect on enzyme activity for β-glucosidase or phosphatase. Sinsabaugh et al. [24] found a negative relationship between soil pH and activity of these two enzymes. In the present case, the possible negative effect on enzyme activity due to the decrease in soil pH could be offset by the positive effect of increasing soil organic matter content in the treatments with topsoil addition.

Depth of spread topsoil and position on the slope seemed to have no effect on the activity of β-glucosidase or phosphatase. This result may be due to the fact that we measured enzymatic activity in the upper soil layer (10 cm depth), as recommended by other authors [25], [41] who measured enzymatic activities to infer soil functioning in Mediterranean grasslands, as this is where most of the annual species' rizhosphere is located. The short time since the topsoil was spread could explain why no differences were found in enzyme activity on different parts of the slope (upper, middle and lower). Material from upper slope positions that builds up in the lower parts often generates a gradient of soil fertility and moisture, which affects enzyme activity [55] and vegetation. This process had not begun when the samples were taken, although one would expect the gradient to appear over time and be reflected in the form of more enzyme activity in the lower than the upper zone of the fill [56].

As in the case of the activity of β-glucosidase and phosphatase enzymes, soil respiration was greater in the presence of topsoil than in its absence (Fig. 2). However, contrary to the pattern found in enzyme activity, soil respiration increased with the spread topsoil depth, showing greater microbial activity in plots with 30 cm of spread soil than 10 cm. This result might be due to the way soil respiration was measured. The device used to quantify soil respiration measured the CO_2_ produced in the top 10 cm, and also the CO_2_ generated further below by microbial activity during measurement. The greater soil depth in T30 probably implies greater microbial density than the shallow soil layer (T10) and hence higher CO_2_ production per surface unit, even if microbial density decreases with soil depth [57]. Seasonal fluctuations in microbial activity, measured via soil respiration, are mainly due to changes in the soil moisture and temperature conditions [58], [59], as the rainiest months are the periods of greatest activity in disturbed soils [60], [61]. Monthly soil respiration readings confirm this trend, with higher respiration, particularly in the treatments, during periods of mild temperatures and higher rainfall, normally autumn (November) and spring (February to July) in Mediterranean regions.

Monthly levels of soil respiration ranged between 0.038 and 0.464 g CO_2_·m^−2^·h^−1^ in soils with more organic matter, in contrast to 0.050 and 0.255 g CO_2_·m^−2^·h^−1^ recorded in the control plot soils. These levels are similar to those of temperate grasslands (0.050 g CO_2_·m^−2^·h^−1^) [62] and well-drained soils in temperate mixed forests (0.05 and 0.25 g CO_2_·m^−2^·h^−1^) [63], but slightly lower than those of forest soils (0.088 and 0.66 g CO_2_·m^−2^·h^−1^ minimum and maximum respectively) [64] and Mediterranean forests (0.268 and 0.887 g CO_2_·m^−2^·h^−1^) [59]. These differences may be due mainly to the heavy influence of forest tree roots on soil respiration [65], [66], a factor that was absent from the present study.

### Topsoil spreading and vegetation

Erosion control is one of the principal objectives of roadslope restoration. Rapid establishment of plant cover guarantees the stability of such surfaces [67], [68] and is favoured by the application of topsoil [13]. However, this effect was not clear in our study. Contrary to expectations [8], [13], in the spring following the topsoil application, plant cover was greater in the topsoil-free plots than in the others and there were no difference in species richness between treatments. This was basically due to the dominance of a few species: *Polygonum aviculare, Spergularia purpurea* and *Anthemis arvensis*, whose seeds have a persistent character and are present in deep soil levels [69], and thus may have been present on the embankment before topsoil was applied. These species attained higher cover values in plots with poorer soils since they are pioneer colonists, adapted to growth in nutrient-poor environments and less likely to thrive in nutrient-rich soils [70]. This result is congruent with the lower equitability of the control plots.

The embankment experienced a significant influx of seeds from neighbouring areas during summer–autumn 2010 [71]–[73]. This input can exceed the amount originating in the topsoil [8]. Thus, in the second spring, the topsoil plots showed a greater increase in plant cover than the controls and also a significant increase in species richness. Higher species richness may confer greater stability on roadslopes since it is presumed to increase the functionality of the ecosystem [74], broadening species phenology across critical periods. This increase in floristic diversity during the first stages of plant succession when restoring roadslopes has been described by other authors [73], [75]. Generalist species greatly augment species richness through their ability to colonize bare and poor terrain. As these spaces become filled, interspecific competition leads the community to a more stable situation, with greater overall plant cover but lower species diversity, the outcome of competitive exclusion processes [12], [73], [76].

The depth of the spread topsoil does not seem to affect the restoration process significantly with respect to plant cover, species diversity (richness and equitability) or floristic composition. Other studies have described how seedling recruitment does not differ between applications of 10 cm or 30 cm of topsoil [9] and that the density of seedlings emerging from the seed bank is not affected by topsoil depth between 1 and 8 cm [77]. This is probably because seeds buried deeper than 5 cm experience difficulty in germination and seedling emergence [9], [36], [78]. Although other authors have noted an increase in plant cover [14] and lower species richness with increased topsoil depth [79], due to the increased availability of organic matter, nitrogen, phosphorus and available water, these findings are not confirmed by our study. This discrepancy is probably due to the fact that our communities were mainly composed of herbaceous annual plants, while the former studies described restoration in woody and perennial herbaceous communities that can take advantage of deeper fertile layers due to their deeper root systems.

## Conclusions

The present study validates some of the initial hypotheses. Spread topsoil does seem to increase microbial activity and vegetation recovery on newly build road embankments. However, the 30 cm depth of the topsoil spread across the embankments to be restored does not seem to cause significant changes in species richness, equitability and floristic composition in comparison with the 10 cm depth. It is therefore recommended that topsoil should be spread to a depth of no more than 10 cm in order to optimize the use of this resource, enabling organic matter to be saved or used to treat a larger area. Nevertheless, the quality of the added soil relative to the original substrate must be checked for each particular case, especially for organic matter content, an essential soil component for the stimulation of microbial activity and hence plant growth, as highlighted by the findings of this and previous papers.

## Supporting Information

Table S1 **Mean and standard error (SD) of species cover (%) in the different plots and study years.** C =  Control, original substrate, T10  =  topsoil applied to 10 cm depth, T30  =  topsoil applied to 30 cm depth. Only species present in more than 1 quadrat have been listed.
(DOCX)
