# Supplementary material for: Spreading Topsoil Encourages Ecological Restoration on Embankments: Soil Fertility, Microbial Activity and Vegetation Cover
Source: PLoS One. 2014 Jul 1;9(7):e101413. doi: 10.1371/journal.pone.0101413 (PMC4077791; doi:10.1371/journal.pone.0101413)
Supplement: Table S1 — Mean and standard error (SD) of species cover (%) in the different plots and study years. C = Control, original substrate, T10 = topsoil applied to 10 cm depth, T30 = topsoil applied to 30 cm depth. Only species present in more than 1 quadrat have been listed. (DOCX) [file pone.0101413.s001.docx]

Table S1. Mean and standard error (SD) of species cover (%) in the different plots and study years. C= Control, original substrate, T10 = topsoil applied to 10 cm depth, T30 = topsoil applied to 30 cm depth. Only species present in more than 1 quadrat have been listed.

| Year | 2010 | | | | | | 2011 | | | | | |
| --- | --- | --- | --- | --- | --- | --- | --- | --- | --- | --- | --- | --- |
| Treatment | C | | T10 | | T30 | | C | | T10 | | T30 | |
| Species |  | |  | |  | |  | |  | |  | |
|  | Mean | SD | Mean | SD | Mean | SD | Mean | SD | Mean | SD | Mean | SD |
| *Agrostis castellana* Boiss. & Reut. | 0.00 | 0.00 | 0.00 | 0.00 | 0.00 | 0.00 | 0.00 | 0.00 | 0.63 | 2.77 | 0.00 | 0.00 |
| *Alyssum granatense* Boiss. & Reut. | 0.00 | 0.00 | 0.00 | 0.00 | 0.00 | 0.00 | 0.00 | 0.00 | 0.07 | 0.25 | 0.00 | 0.00 |
| *Anacyclus clavatus* (Desf.) Pers. | 0.00 | 0.00 | 0.00 | 0.00 | 0.00 | 0.00 | 19.37 | 14.01 | 9.40 | 9.20 | 7.20 | 6.71 |
| *Anagallis arvensis* L. | 0.00 | 0.00 | 0.00 | 0.18 | 0.00 | 0.00 | 0.00 | 0.00 | 1.50 | 6.04 | 5.33 | 14.20 |
| *Andryala integrifolia* L. | 0.00 | 0.00 | 0.03 | 0.00 | 0.17 | 0.93 | 0.47 | 1.87 | 0.17 | 0.38 | 0.13 | 0.43 |
| *Anthemis arvensis* L. | 9.37 | 9.58 | 0.00 | 2.96 | 0.45 | 1.21 | 0.23 | 0.97 | 0.70 | 3.65 | 0.60 | 2.03 |
| *Aphanes microcarpa* (Boiss. & Reut.) Rothm. | 0.03 | 0.18 | 0.83 | 1.28 | 0.14 | 0.35 | 0.00 | 0.00 | 0.57 | 1.01 | 0.87 | 1.87 |
| *Arabidopsis thaliana* (L.) Heynh. in Holl & Heynh. | 0.03 | 0.18 | 0.47 | 3.04 | 1.14 | 1.33 | 0.20 | 0.92 | 0.80 | 1.35 | 0.70 | 1.06 |
| *Asterolinon linum-stellatum* (L.) Duby in DC. | 0.00 | 0.00 | 2.47 | 0.00 | 0.03 | 0.19 | 0.00 | 0.00 | 0.07 | 0.25 | 0.17 | 0.53 |
| *Astragalus pelecinus* (L.) Barneby | 0.00 | 0.00 | 0.00 | 0.00 | 0.03 | 0.19 | 0.87 | 2.19 | 0.00 | 0.00 | 0.07 | 0.37 |
| *Brassica barrelieri* (L.) Janka | 2.50 | 5.86 | 0.00 | 0.00 | 0.17 | 0.93 | 3.70 | 5.95 | 2.40 | 5.96 | 1.73 | 3.69 |
| *Bromus hordeaceus* L. | 0.03 | 0.18 | 0.00 | 0.00 | 0.03 | 0.19 | 0.57 | 1.14 | 1.13 | 2.27 | 1.60 | 2.49 |
| *Bromus madritensis* L. | 0.03 | 0.18 | 0.00 | 0.00 | 0.31 | 0.71 | 1.33 | 2.75 | 5.00 | 6.21 | 4.60 | 4.97 |
| *Bromus rubens* L. | 0.17 | 0.91 | 0.00 | 0.00 | 0.00 | 0.00 | 0.03 | 0.18 | 0.00 | 0.00 | 0.13 | 0.43 |
| *Bromus sterilis* L. | 0.00 | 0.00 | 0.00 | 2.27 | 0.52 | 0.83 | 0.00 | 0.00 | 0.00 | 0.00 | 0.17 | 0.91 |
| *Bromus tectorum* L. | 0.00 | 0.00 | 1.60 | 0.00 | 0.31 | 1.07 | 1.10 | 2.63 | 1.97 | 3.65 | 4.73 | 7.24 |
| *Calendula arvensis* L. | 0.00 | 0.00 | 0.00 | 0.00 | 0.00 | 0.00 | 0.00 | 0.00 | 0.07 | 0.37 | 0.10 | 0.40 |
| *Cardamine hirsuta* L. | 0.00 | 0.00 | 0.00 | 0.68 | 0.10 | 0.56 | 0.00 | 0.00 | 0.00 | 0.00 | 0.00 | 0.00 |
| *Carlina corymbosa* L. | 0.00 | 0.00 | 0.23 | 0.00 | 0.00 | 0.00 | 0.67 | 2.54 | 0.00 | 0.00 | 0.00 | 0.00 |
| *Centranthus calcitrapae* (L.) Dufr. | 0.00 | 0.00 | 0.00 | 0.18 | 0.00 | 0.00 | 0.03 | 0.18 | 0.07 | 0.25 | 0.10 | 0.40 |
| *Cerastium glomeratum* Thuill. | 0.00 | 0.00 | 0.03 | 2.76 | 0.34 | 0.67 | 0.33 | 0.99 | 1.37 | 1.65 | 1.30 | 1.49 |
| *Chondrilla juncea* L. | 0.00 | 0.00 | 1.67 | 0.00 | 0.03 | 0.19 | 0.07 | 0.25 | 0.33 | 1.83 | 0.00 | 0.00 |
| *Conyza sp* | 0.47 | 1.83 | 0.00 | 0.25 | 0.45 | 1.38 | 0.03 | 0.18 | 0.00 | 0.00 | 0.07 | 0.25 |
| *Corynephorus divaricatus (Pourr.) Breistr.* | 0.00 | 0.00 | 0.07 | 0.18 | 0.10 | 0.41 | 0.03 | 0.18 | 0.73 | 1.34 | 0.30 | 0.60 |
| *Crassula tillaea Lest.-Garl.* | 0.07 | 0.25 | 0.03 | 1.33 | 0.14 | 0.35 | 0.00 | 0.00 | 0.10 | 0.31 | 0.03 | 0.18 |
| *Crepis capillaris* (L.) Wallr. | 0.00 | 0.00 | 0.57 | 0.00 | 0.38 | 1.86 | 0.37 | 1.03 | 0.57 | 1.33 | 2.17 | 4.53 |
| *Crepis vesicaria* L. | 0.03 | 0.18 | 0.00 | 0.00 | 0.00 | 0.00 | 0.40 | 1.85 | 1.03 | 2.94 | 1.33 | 3.10 |
| *Cynodon dactylon* (L.) Pers. | 0.00 | 0.00 | 0.00 | 0.00 | 0.00 | 0.00 | 0.50 | 1.83 | 0.00 | 0.00 | 1.70 | 5.30 |
| *Dactylis glomerata* L. | 0.00 | 0.00 | 0.00 | 0.31 | 0.07 | 0.26 | 0.00 | 0.00 | 0.83 | 2.65 | 1.40 | 4.13 |
| *Diplotaxis erucoides* (L.) DC. | 1.33 | 3.06 | 0.10 | 0.00 | 0.00 | 0.00 | 0.00 | 0.00 | 0.00 | 0.00 | 0.00 | 0.00 |
| *Echium plantagineum* L. | 0.67 | 3.65 | 0.00 | 0.00 | 0.00 | 0.00 | 2.27 | 5.50 | 0.03 | 0.18 | 0.00 | 0.00 |
| *Epilobium brachycarpum* C. Presl | 0.93 | 2.96 | 0.00 | 1.85 | 0.03 | 0.19 | 8.30 | 7.75 | 8.83 | 7.20 | 7.97 | 6.09 |
| *Erodium cicutarium* (L.) L´Her. | 0.00 | 0.00 | 0.43 | 0.00 | 0.10 | 0.56 | 0.03 | 0.18 | 2.10 | 6.22 | 0.90 | 3.24 |
| *Eryngium campestre* L. | 0.00 | 0.00 | 0.00 | 0.00 | 0.34 | 1.86 | 0.67 | 3.65 | 0.00 | 0.00 | 0.03 | 0.18 |
| *Filago pyramidata* L. | 0.00 | 0.00 | 0.00 | 0.00 | 0.03 | 0.19 | 0.00 | 0.00 | 0.53 | 0.97 | 1.30 | 3.72 |
| *Fumaria sp* | 0.00 | 0.00 | 0.00 | 0.48 | 0.38 | 0.86 | 0.00 | 0.00 | 0.00 | 0.00 | 0.00 | 0.00 |
| *Galium parisiense* L. | 0.00 | 0.00 | 0.20 | 0.53 | 0.24 | 0.64 | 0.10 | 0.31 | 0.67 | 1.06 | 0.83 | 1.32 |
| *Herniaria glabra* L. | 0.17 | 0.91 | 0.30 | 0.18 | 0.28 | 1.49 | 0.00 | 0.00 | 0.00 | 0.00 | 0.00 | 0.00 |
| *Herniaria hirsuta* L. | 0.07 | 0.25 | 0.03 | 0.00 | 0.00 | 0.00 | 2.03 | 4.59 | 0.67 | 3.65 | 0.40 | 1.85 |
| *Hymenocarpos cornicina* (L.) Vis. | 0.00 | 0.00 | 0.00 | 0.00 | 0.00 | 0.00 | 4.77 | 6.63 | 2.20 | 3.60 | 2.10 | 2.68 |
| *Hymenocarpos lotoides* (L.) Vis. | 0.90 | 3.08 | 0.00 | 0.00 | 0.03 | 0.19 | 4.37 | 7.38 | 0.87 | 2.30 | 1.90 | 3.81 |
| *Hypochaeris glabra* L. | 0.00 | 0.00 | 0.00 | 1.46 | 0.10 | 0.56 | 1.33 | 3.82 | 1.67 | 4.03 | 1.00 | 2.08 |
| *Jasione montana* L. | 0.07 | 0.25 | 0.27 | 1.28 | 0.17 | 0.38 | 0.03 | 0.18 | 1.20 | 2.96 | 0.70 | 2.00 |
| *Juncus bufonius* L. | 0.00 | 0.00 | 0.50 | 1.17 | 0.14 | 0.44 | 0.00 | 0.00 | 0.00 | 0.00 | 0.03 | 0.18 |
| *Lactuca serriola* L. | 0.00 | 0.00 | 0.53 | 0.00 | 0.00 | 0.00 | 0.17 | 0.91 | 0.00 | 0.00 | 0.83 | 2.96 |
| *Lathyrus angulatus* L. | 0.00 | 0.00 | 0.00 | 0.00 | 0.34 | 1.86 | 4.23 | 6.49 | 1.70 | 3.47 | 1.70 | 2.28 |
| *Leontodon taraxacoides* (Vill.) Marat | 0.00 | 0.00 | 0.00 | 0.59 | 0.03 | 0.19 | 0.17 | 0.91 | 1.03 | 2.65 | 0.63 | 2.04 |
| *Linaria spartea* (L.) Chaz. | 0.00 | 0.00 | 0.17 | 0.00 | 0.24 | 0.79 | 0.00 | 0.00 | 0.07 | 0.25 | 0.17 | 0.46 |
| *Logfia gallica* (L.) Coss. & Germ. | 0.20 | 0.41 | 0.00 | 0.00 | 0.00 | 0.00 | 0.07 | 0.25 | 0.07 | 0.25 | 0.07 | 0.25 |
| *Logfia minima* (Sm.) Dumort. | 0.03 | 0.18 | 0.00 | 0.00 | 0.00 | 0.00 | 0.03 | 0.18 | 0.33 | 0.71 | 0.13 | 0.43 |
| *Lolium rigidum* Gaudin | 0.20 | 0.92 | 0.00 | 0.00 | 0.00 | 0.00 | 0.00 | 0.00 | 0.50 | 2.74 | 0.00 | 0.00 |
| *Mibora minima* (L.) Desv. | 0.20 | 0.66 | 0.00 | 0.00 | 0.03 | 0.19 | 0.07 | 0.25 | 0.53 | 1.31 | 0.67 | 1.30 |
| *Minuartia sp* | 0.03 | 0.18 | 0.00 | 0.00 | 0.00 | 0.00 | 0.00 | 0.00 | 0.03 | 0.18 | 0.03 | 0.18 |
| *Myosotis personii* Rouy | 0.00 | 0.00 | 0.00 | 2.54 | 0.14 | 0.74 | 0.00 | 0.00 | 0.00 | 0.00 | 0.00 | 0.00 |
| *Myosotis ramosissima* Rochel in Schultes | 0.00 | 0.00 | 0.77 | 0.25 | 0.00 | 0.00 | 0.17 | 0.38 | 0.40 | 1.30 | 0.30 | 0.95 |
| *Myosotis stricta* Roemer & Schultes | 0.00 | 0.00 | 0.07 | 1.83 | 0.10 | 0.56 | 0.00 | 0.00 | 0.00 | 0.00 | 0.00 | 0.00 |
| *Ornithopus compressus* L. | 0.57 | 2.03 | 0.33 | 0.91 | 0.03 | 0.19 | 7.37 | 6.73 | 4.03 | 4.79 | 4.00 | 3.88 |
| *Papaver argemone* L. | 0.03 | 0.18 | 0.17 | 0.31 | 0.03 | 0.19 | 0.00 | 0.00 | 0.00 | 0.00 | 0.00 | 0.00 |
| *Papaver dubium* L. | 0.23 | 0.63 | 0.10 | 1.83 | 1.03 | 2.71 | 0.00 | 0.00 | 0.00 | 0.00 | 0.00 | 0.00 |
| *Papaver rhoeas* L. | 0.00 | 0.00 | 0.33 | 0.00 | 0.00 | 0.00 | 0.00 | 0.00 | 0.03 | 0.18 | 0.07 | 0.25 |
| *Plantago lagopus* L. | 0.03 | 0.18 | 0.00 | 0.00 | 0.00 | 0.00 | 0.17 | 0.91 | 0.00 | 0.00 | 0.00 | 0.00 |
| *Polygonum aviculare* L. | 17.37 | 10.67 | 0.00 | 4.44 | 1.10 | 2.94 | 0.00 | 0.00 | 0.00 | 0.00 | 0.00 | 0.00 |
| *Psilurus incurvus* (Gouan) Schinz & Thell. | 0.00 | 0.00 | 2.37 | 0.00 | 0.00 | 0.00 | 1.67 | 4.36 | 0.00 | 0.00 | 0.00 | 0.00 |
| *Raphanus raphanistrum* L. | 0.00 | 0.00 | 0.00 | 0.00 | 0.00 | 0.00 | 0.07 | 0.37 | 0.03 | 0.18 | 0.00 | 0.00 |
| *Rumex acetosella* L. | 4.23 | 4.14 | 0.00 | 4.11 | 2.31 | 4.24 | 11.83 | 9.66 | 20.77 | 18.31 | 26.50 | 15.09 |
| *Sanguisorba minor* Scop | 0.00 | 0.00 | 2.87 | 0.00 | 0.03 | 0.19 | 0.17 | 0.91 | 2.43 | 6.63 | 0.73 | 2.85 |
| *Senecio gallicus* Chaix | 0.00 | 0.00 | 0.00 | 0.18 | 0.17 | 0.60 | 0.00 | 0.00 | 0.00 | 0.00 | 0.23 | 0.94 |
| *Senecio vulgaris* L. | 0.10 | 0.31 | 0.03 | 0.55 | 0.07 | 0.37 | 0.70 | 1.53 | 0.20 | 0.48 | 0.17 | 0.38 |
| *Sesamoides interrupta* (Boreau) G. López | 1.17 | 1.39 | 0.10 | 0.00 | 0.38 | 1.29 | 3.90 | 5.74 | 0.33 | 1.83 | 0.57 | 2.75 |
| *Spergula arvensis* L. | 0.13 | 0.51 | 0.00 | 1.30 | 0.93 | 2.60 | 0.40 | 1.10 | 4.73 | 6.27 | 4.60 | 5.37 |
| *Spergula pentandra* L. | 0.00 | 0.00 | 0.40 | 5.51 | 0.00 | 0.00 | 0.00 | 0.00 | 0.07 | 0.37 | 0.63 | 1.56 |
| *Spergularia purpurea* (Pers.) D. Don | 19.37 | 13.07 | 1.30 | 9.38 | 2.48 | 5.21 | 1.93 | 3.37 | 7.17 | 9.26 | 6.33 | 6.05 |
| *Taeniatherum caput-medusae* (L.) Nevski | 0.00 | 0.00 | 4.57 | 0.00 | 0.00 | 0.00 | 1.70 | 4.32 | 0.03 | 0.18 | 0.00 | 0.00 |
| *Taraxacum officinale* Weber | 0.00 | 0.00 | 0.00 | 0.00 | 0.00 | 0.00 | 0.07 | 0.25 | 0.33 | 1.83 | 0.00 | 0.00 |
| *Teesdalia coronopifolia* (J.P. Bergeret) Thell. | 0.00 | 0.00 | 0.00 | 0.51 | 0.00 | 0.00 | 0.00 | 0.00 | 0.37 | 0.61 | 0.30 | 0.70 |
| *Tolpis barbata* (L.) Gaertner | 0.00 | 0.00 | 0.13 | 0.00 | 0.00 | 0.00 | 0.00 | 0.00 | 0.10 | 0.31 | 0.30 | 0.95 |
| *Trifolium angustifolium* L. | 0.00 | 0.00 | 0.00 | 0.00 | 0.00 | 0.00 | 0.00 | 0.00 | 0.17 | 0.91 | 0.13 | 0.73 |
| *Trifolium arvense* L. | 2.13 | 5.78 | 0.00 | 0.00 | 0.07 | 0.37 | 15.03 | 11.22 | 2.70 | 3.49 | 3.60 | 4.60 |
| *Trifolium campestre* Schreb. in Sturm | 0.10 | 0.55 | 0.00 | 0.00 | 0.00 | 0.00 | 0.03 | 0.18 | 0.07 | 0.25 | 0.07 | 0.25 |
| *Trifolium cernuum* Brot. | 0.00 | 0.00 | 0.00 | 0.00 | 0.00 | 0.00 | 1.07 | 4.02 | 0.07 | 0.25 | 0.00 | 0.00 |
| *Trifolium cherleri* L. | 0.27 | 0.98 | 0.00 | 1.27 | 0.10 | 0.56 | 9.23 | 8.52 | 9.83 | 6.88 | 10.30 | 5.98 |
| *Trifolium gemellum* Pourr. ex Willd. | 0.00 | 0.00 | 0.33 | 0.00 | 0.00 | 0.00 | 0.00 | 0.00 | 0.03 | 0.18 | 0.17 | 0.91 |
| *Trifolium hirtum* All. | 0.33 | 1.83 | 0.00 | 0.00 | 0.00 | 0.00 | 2.20 | 4.41 | 0.20 | 0.92 | 0.87 | 2.65 |
| *Trifolium scabrum* L. | 0.33 | 1.09 | 0.00 | 0.00 | 0.00 | 0.00 | 0.00 | 0.00 | 0.00 | 0.00 | 0.00 | 0.00 |
| *Trifolium subterraneum* L*.* | 0.00 | 0.00 | 0.00 | 0.00 | 0.00 | 0.00 | 0.33 | 1.83 | 1.13 | 2.45 | 1.10 | 3.07 |
| *Trifolium suffocatum* L. | 0.00 | 0.00 | 0.00 | 0.40 | 0.00 | 0.00 | 0.00 | 0.00 | 0.00 | 0.00 | 0.00 | 0.00 |
| *Trifolium tomentosum* L. | 0.00 | 0.00 | 0.10 | 0.00 | 0.00 | 0.00 | 0.57 | 2.75 | 0.00 | 0.00 | 0.10 | 0.55 |
| *Valerianella coronata* (L.) DC. in Lam. & DC. | 0.00 | 0.00 | 0.00 | 0.00 | 0.00 | 0.00 | 0.23 | 0.63 | 0.03 | 0.18 | 0.10 | 0.31 |
| *Veronica arvensis* L. | 0.00 | 0.00 | 0.00 | 0.00 | 0.00 | 0.00 | 0.03 | 0.18 | 0.00 | 0.00 | 0.03 | 0.18 |
| *Veronica verna* L. | 0.17 | 0.91 | 0.00 | 0.18 | 0.07 | 0.26 | 0.00 | 0.00 | 0.10 | 0.31 | 0.17 | 0.59 |
| *Vicia lathyroides* L. | 0.00 | 0.00 | 0.03 | 0.00 | 0.00 | 0.00 | 0.00 | 0.00 | 0.53 | 1.38 | 0.17 | 0.59 |
| *Vicia lutea* L. | 0.00 | 0.00 | 0.00 | 0.00 | 0.00 | 0.00 | 0.00 | 0.00 | 0.03 | 0.18 | 0.37 | 1.83 |
| *Vicia sativa* L. | 0.00 | 0.00 | 0.00 | 0.00 | 0.00 | 0.00 | 0.00 | 0.00 | 0.47 | 1.28 | 0.20 | 0.48 |
| *Viola kitaibeliana* Schult. in Roem. & Schult. | 0.00 | 0.00 | 0.00 | 0.71 | 0.14 | 0.58 | 0.07 | 0.25 | 0.50 | 1.28 | 0.33 | 0.55 |
| *Vulpia ciliata* Dumort. | 0.17 | 0.53 | 0.33 | 0.00 | 0.07 | 0.37 | 0.63 | 1.22 | 0.00 | 0.00 | 0.17 | 0.53 |
| *Vulpia muralis* (Kunth) Nees | 0.00 | 0.00 | 0.00 | 0.57 | 0.00 | 0.00 | 0.77 | 2.13 | 0.17 | 0.91 | 0.00 | 0.00 |
| *Vulpia myuros* (L.) C.C.Gmel. | 0.23 | 0.97 | 0.13 | 0.00 | 0.00 | 0.00 | 0.20 | 0.48 | 1.43 | 4.06 | 1.40 | 3.32 |
| *Xolantha guttata* (L.) Raf. | 0.03 | 0.18 | 0.00 | 0.31 | 0.17 | 0.47 | 0.03 | 0.18 | 0.00 | 0.00 | 0.07 | 0.25 |
